# Extended half-life target module for sustainable UniCAR T-cell treatment of STn-expressing cancers

**DOI:** 10.1186/s13046-020-01572-4

**Published:** 2020-05-05

**Authors:** Liliana R. Loureiro, Anja Feldmann, Ralf Bergmann, Stefanie Koristka, Nicole Berndt, Domokos Máthé, Nikolett Hegedüs, Krisztián Szigeti, Paula A. Videira, Michael Bachmann, Claudia Arndt

**Affiliations:** 1 Helmholtz-Zentrum Dresden-Rossendorf (HZDR), Institute of Radiopharmaceutical Cancer Research, Bautzner Landstrasse 400, 01328 Dresden, Germany; 2 National Center for Tumor Diseases (NCT), Dresden, Germany; German Cancer Research Center (DKFZ), Heidelberg, Germany; Faculty of Medicine and University Hospital Carl Gustav Carus, Technische Universität Dresden, Dresden, Germany; Helmholtz-Zentrum Dresden-Rossendorf (HZDR), Dresden, Germany; 3 Department of Biophysics and Radiation Biology, Faculty of Medicine, Semmelweis University, Budapest, Hungary; 4 UCIBIO, Departamento Ciências da Vida, Faculdade de Ciências e Tecnologia, Universidade NOVA de Lisboa, Caparica, Portugal; 5 German Cancer Consortium (DKTK), partner site Dresden and German Cancer Research Center (DKFZ), Heidelberg, Germany; 6 Tumor Immunology, University CancerCenter (UCC), University Hospital Carl Gustav Carus Dresden, Technische Universität Dresden, Dresden, Germany

**Keywords:** Immunotherapy, UniCAR T-cells, IgG4-based TM, Sialyl-Tn (STn)

## Abstract

**Background:**

Adapter chimeric antigen receptor (CAR) approaches have emerged has promising strategies to increase clinical safety of CAR T-cell therapy. In the UniCAR system, the safety switch is controlled via a target module (TM) which is characterized by a small-size and short half-life. The rapid clearance of these TMs from the blood allows a quick steering and self-limiting safety switch of UniCAR T-cells by TM dosing. This is mainly important during onset of therapy when tumor burden and the risk for severe side effects are high. For long-term UniCAR therapy, the continuous infusion of TMs may not be an optimal setting for the patients. Thus, in later stages of treatment, single infusions of TMs with an increased half-life might play an important role in long-term surveillance and eradication of residual tumor cells. Given this, we aimed to develop and characterize a novel TM with extended half-life targeting the tumor-associated carbohydrate sialyl-Tn (STn).

**Methods:**

The extended half-life TM is composed of the STn-specific single-chain variable fragment (scFv) and the UniCAR epitope, fused to the hinge region and Fc domain of a human immunoglobulin 4 (IgG4) antibody. Specific binding and functionality of the αSTn-IgG4 TM as well as pharmacokinetic features were assessed using in vitro and in vivo assays and compared to the already established small-sized αSTn TM.

**Results:**

The novel αSTn-IgG4 TM efficiently activates and redirects UniCAR T-cells to STn-expressing tumors in a target-specific and TM-dependent manner, thereby promoting the secretion of proinflammatory cytokines and tumor cell lysis in vitro and in experimental mice. Moreover, PET-imaging results demonstrate the specific enrichment of the αSTn-IgG4 TM at the tumor site, while presenting a prolonged serum half-life compared to the short-lived αSTn TM.

**Conclusion:**

In a clinical setting, the combination of TMs with different formats and pharmacokinetics may represent a promising strategy for retargeting of UniCAR T-cells in a flexible, individualized and safe manner at particular stages of therapy. Furthermore, as these molecules can be used for in vivo imaging, they pose as attractive candidates for theranostic approaches.

## Background

**Fig. 1:**
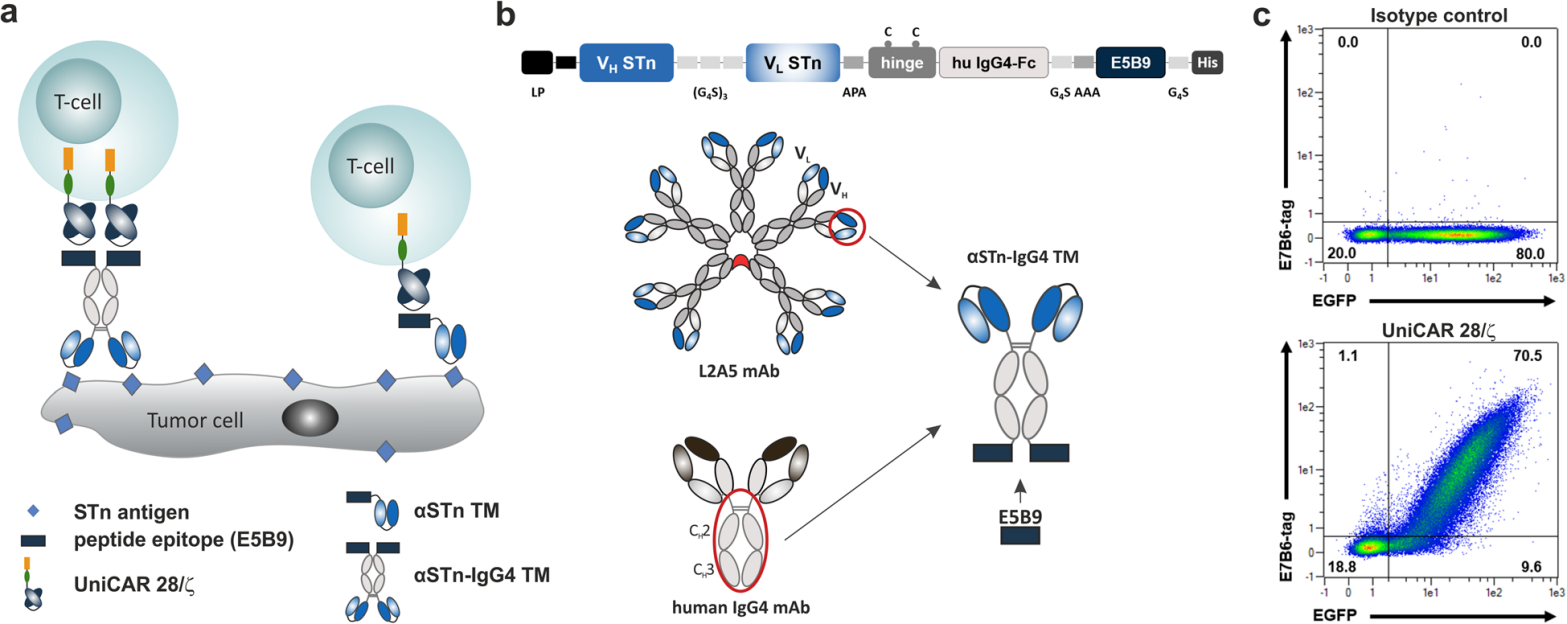
Illustrative representation of the UniCAR system and the αSTn-IgG4 TM construct. **a** The UniCAR system can be combined with different TM formats, namely scFv- and IgG4-based TMs, to specifically redirect UniCAR T-cells to tumor cells via the E5B9 epitope. UniCAR T-cells are genetically engineered to express the humanized scFv derived from the anti-La mAb 5B9 fused to the transmembrane domain of human CD28 and intracellular signaling domains of CD28 and CD3ζ. **b** Detailed representation of the αSTn-IgG4 TM construct, comprising the anti-STn scFv (L2A5) arranged in V_H_ - V_L_ orientation linked by a (G_4_S)_3_ peptide, the hinge and Fc region of the human IgG4 molecule, and the E5B9 epitope. After expression the αSTn-IgG4 TM will form homodimers due to formation of disulfide bridges between the cysteine residues present in the IgG4 hinge region. **c** In order to confirm UniCAR expression, genetically modified T-cells were stained with isotype control (upper panel) or anti-La mAb 7B6 (lower panel). Specific binding was detected using a PE-labeled anti-mouse-IgG mAb. Density plots show percentage of CAR surface expression (E7B6) versus expression of co-translated EGFP protein marker for one representative donor. G, glycine; S, serine; A, alanine; P, proline; C, cysteine

CAR T-cell therapies are moving at a breakneck pace, demonstrating remarkable clinical success particularly in the treatment of blood malignancies [1]. Nevertheless, some challenges still hinder their broad clinical application, mainly related to on-target/off-tumor toxicity, cytokine release syndrome and tumor escape variants [2–5]. Several approaches have been pursued in recent years, aiming an increased safety by reducing the risk for these recurrently reported side effects. Among them, the UniCAR system developed by our group may fulfill the requirements to improve specificity and safety of CAR T-cell therapies [6]. In contrast to conventional CAR T-cell approaches, UniCAR T-cells are not directed to a tumor antigen but alternatively recognize the peptide epitope E5B9. This epitope is derived from the nuclear protein La/SS-B, which is not accessible on the surface of intact living cells. UniCAR T-cells are therefore engineered to express universal receptors composed of the extracellular binding domain against the epitope E5B9, the transmembrane domain derived from CD28 as well as the intracellular signaling domains of CD28 and CD3ζ. The cross-linkage between UniCAR effector cells and target cells is mediated via a target module (TM) composed of a binding moiety against a specific tumor antigen fused to the E5B9 epitope (Fig. 1a) [7, 8]. This in turn leads to the specific activation of the otherwise inert UniCAR T-cells and allows a controlled on/off switch of the UniCAR T-cells in case severe side effects are observed. Furthermore, multiple targeting strategies can be considered given that TMs can be easily constructed and interchanged using different formats and specificities [8–12].

A repertoire of small-sized TMs directed against various tumor-associated antigens has been developed in our group. Given their short half-life, these TMs are rapidly eliminated from the blood [8–12], which allows a quick control over UniCAR T-cell activity in case side effects occur. Nonetheless, continuous administration is required during therapy to maintain effective TM concentrations and perpetuate tumor cell killing by UniCAR T-cells. Considering long-term treatment, a prolonged activation of UniCAR T-cells to eradicate residual tumor cells as well as reduction of TM infusions may be desirable for an improved therapeutic approach and patient care at later phases of therapy. Therefore, in this work we aimed to establish an extended half-life TM that could present a promising tool for late-stage UniCAR T-cell therapy while preserving the switchable safety features of the UniCAR system. Within the collection of small-sized TMs developed, a scFv-based TM directed against the tumor-associated carbohydrate antigen sialyl-Tn (STn) was proven to efficiently and specifically redirect UniCAR T-cells to STn-expressing cancer cells [12]. This antigen is widely overexpressed in several types of human carcinomas, such as gastric [13], colorectal [14], ovarian [15], breast [16] and bladder [17] carcinomas. Given that STn expression modulates a malignant phenotype and is particularly associated with cancer aggressiveness, metastasis and poor prognosis [18–20], increasing attention has been recently drawn towards this tumor antigen. Building upon this TM, a novel αSTn-IgG4 TM based on the Fc region of immunoglobulin 4 (IgG4) antibodies was developed and assessed for its capability to redirect UniCAR T-cells to STn-expressing cancer cells both in vitro and in vivo.

## Methods

### Cell lines

STn expression is absent in the wild-type (WT) human bladder cancer cell line MCR and in the WT human breast cancer cell line MDA-MB-231 when cultured in vitro. Therefore, MCR and MDA-MB-231 cell lines expressing the STn antigen were generated as previously reported [17, 21]. The murine 3T3 (ATCC CRL-1658) fibroblast cells were used for production of αSTn and αSTn-IgG4 TMs. Human embryonic kidney cells HEK293T (ATCC CRL-11268) served as virus producing cells. The MDA-MB-231 STn^+^ cell line was additionally transduced with an open reading frame encoding the firefly luciferase as previously described [22] and termed MDA-MB-231 STn^+^ Luc. All cell lines were cultured in Dulbecco’s modified Eagle medium (DMEM), supplemented with 10% (v/v) FBS, 2 mM L-glutamine, 100 U/ml of penicillin and 100 μg/ml of streptomycin (Biochrom) and maintained at 37°C in a humidified atmosphere of 5% CO_2_. For routine cell culture and in preparation for experiments, cells were harvested from the culture flasks using Trypsin-EDTA diluted in sterile phosphate-buffered saline (PBS).

### Construction, expression and purification of recombinant antibodies

The αSTn TM was produced as described previously [12]. For in silico construction of the αSTn-IgG4 TM, the variable light (V_L_) and heavy (V_H_) chains of the monoclonal antibody (mAb) L2A5 were arranged in a V_H_-V_L_ orientation and connected by a (Gly_4_Ser)_3_ linker. The variable regions were further linked downstream to the hinge and Fc region (C_H_2-C_H_3) derived from a human IgG4 antibody. The UniCAR tag (E5B9) followed by a 6xHis tag was fused to the C-terminus of this molecule. An Igκ leader peptide (LP) sequence was fused to the N-terminus to promote secretion of the TM into the cell culture supernatant. The final sequence was cloned into the vector p6NST50 and used for generation of stable TM-producing 3T3 cell lines. Recombinant TMs were purified from cell culture supernatant via protein A affinity chromatography according to manufacturer’s instructions (Protein A HP Spin Trap, Sigma-Aldrich) followed by overnight dialysis in PBS (Biochrom). Finally, SDS–PAGE and immunoblotting were used to assess potential contaminants and to determine protein concentration as previously described [23, 24].

### Isolation, maintenance and lentiviral transduction of human T-cells with UniCAR vectors

Primary human T-cells were isolated from peripheral blood mononuclear cells (PBMCs) of buffy coats (supplied by German Red Cross, Dresden, Germany), transduced with UniCAR vectors, sorted and cultured as described previously [23]. Cloning of UniCAR vectors and production of UniCAR T-cells have been also described before [23, 25]. UniCAR vectors include the signaling UniCAR CD28/ζ construct, which contains the co-stimulatory CD28 and activating CD3ζ domains; the UniCAR Stop construct that lacks any intracellular signaling domains; and the UniCAR vector control, encoding only for the EGFP marker protein.

### High-performance liquid chromatography

Purity of the purified TMs was assessed using size exclusion high-performance liquid chromatography (SE-HPLC) as described by Albert et al [10].

### Flow-cytometry analysis

Specific binding of αSTn or αSTn-IgG4 TM to STn-expressing cancer cells and respective equilibrium dissociation constant (*K*_D_) values were determined using flow cytometry. Shortly, 3×10^5^ STn^+^ or WT (STn^−^) cancer cells were incubated for 1 h with 10 ng/μl or increasing concentrations of TM, followed by a washing step and subsequent incubation with mouse anti-La (5B9) mAb for 30 min. Signal was detected using the PE-conjugated goat anti-mouse IgG mAb (Beckmann Coulter) after 30 min of incubation. In order to verify UniCAR expression on the surface of T-cells, the primary anti-La mAb 7B6 and secondary PE-labeled goat anti-mouse-IgG mAb (Beckmann Coulter) were used as described previously [25]. All incubation steps were performed at 4 °C in the dark. Stained cells were analyzed using a MACSQuant^®^ Analyzer and MACSQuantify^®^ software (Miltenyi Biotec). Based on the relative median fluorescence intensity (MFI) of stained cells, a binding curve was obtained and *K*_D_ values were calculated using the nonlinear regression curve fit (95% confidence interval) with GraphPad Prism 7 software (GraphPad Software Inc.).

### Cytokine-release assay

To determine the levels of secreted cytokines, cell-free supernatants from the co-culture of target cells and UniCAR T-cells in the presence or absence of TM were harvest after 24 h incubation. Cytokine concentrations were quantified using the MACSPlex Cytokine 12 Kit (Miltenyi Biotec) according to the manufacturer’s instructions.

### Cell-mediated cytotoxicity assay

Cancer cell lysis mediated by UniCAR T-cells was assessed in vitro by standard chromium-release assay [26]. Briefly, ^51^Cr-labeled STn^+^ cancer cells were co-cultured with the genetically modified UniCAR T-cells in the presence or absence of TMs for 24 h. An effector to target cell (E:T) ratio of 5:1 was used. Different concentrations of TM were applied to calculate the half maximal effective concentration (EC_50_) value.

### TM radiolabeling

All solvents were purchased from commercial sources (Sigma−Aldrich, Fluka, VWR, Fisher Scientific). The no carrier added (NCA) [^64^Cu]Cu^2+^ was produced at the Helmholtz-Zentrum Dresden-Rossendorf on a TR-Flex (Advanced Cyclotron Systems Inc.) by ^64^Ni(p,n)^64^Cu nuclear reaction and prepared as reported previously [27]. For labeling of the αSTn and αSTn-IgG4 TMs with ^64^Cu, [^64^Cu]CuCl_2_ (200 MBq in 0.01 M HCl, 0.3 M NH_4_OAc, pH 5.0) was added to 9.2 ± 6.7 nmol of the TMs and tempered at 38 °C for 30 min. Labeling yield and radiochemical purity were determined using radio thin-layer chromatography (radio-ITLC). The radio-ITLC was carried out on SG stripes (Merck) using 0.1 M citrate, 0.01 M EDTA. The developed chromatograms were analyzed by autoradiography using the In-vivo Multispectral Imaging System (Bruker).

### In vivo experiments using NOD-SCID mice and bioluminescence imaging

Non-obese diabetic (NOD)-severe combined immune-deficient (SCID) female mice at 6 weeks of age were purchased from Janvier Labs (NOD.CB17-*Prkdc*^scid^/Rj). All mice used throughout these experiments were healthy and not involved in previous procedures. Health status of mice was monitored daily by husbandry staff. Mice were euthanized during experiment if they showed hunched abnormal posture, impaired mobility, rough coat, or paralysis. At the end of all experiments, animals were euthanized using carbon dioxide inhalation and cervical dislocation. All animal procedures were approved by the university committee for animal welfare.

To assess in vivo functionality of αSTn and αSTn-IgG4 TMs, optical imaging analysis was performed in which 0.5×10^6^ UniCAR T-cells were mixed with 1×10^6^ MDA-MB-231 STn^+^ Luc cells in the presence or absence of 300 pmol of αSTn or αSTn-IgG4 TM. The corresponding mixtures were subcutaneously injected in the right flank of NOD-SCID mice. Mice were anesthetized using 10% (v/v) desflurane (Baxter) and maintained with 8% (v/v) desflurane inhalation in 30% (v/v) oxygen in order to perform luminescence imaging. Subsequently, mice were injected intraperitoneally with 200 μL of D-luciferin in PBS at a final concentration of 15 mg/ml (Thermo Fisher Scientific). X-ray and luminescence images were acquired 5–10 min after luciferin injection. Bioluminescence imaging was performed at day 0, 1 and 3 using In-vivo Multispectral Imaging System (Bruker). Images were analyzed using the MI 5.3 and MS 1.3 software (Bruker). Quantitative analysis was performed assessing net luminescence intensities of the tumors (photons/s/mm^2^).

### Positron emission tomography (PET) imaging of tumor xenograft models

PET imaging was performed to assess pharmacokinetics and specific accumulation of radiolabeled αSTn TMs ([^64^Cu]Cu-NODAGA-αSTn and [^64^Cu]Cu-NODAGA-αSTn-IgG4 TM) at the tumor site. Tumor induction was achieved by subcutaneous injection of a suspension containing 2×10^6^ MDA-MB-231 STn^+^ Luc cells in PBS into the right shoulder of the mice. Mice were kept under the above-mentioned conditions for 2 to 3 weeks to allow tumor growth. Thereafter, MDA-MB-231 STn^+^ Luc tumor-bearing mice were anesthetized as previously described and 0.7 nmol of [^64^Cu]Cu-NODAGA-αSTn or [^64^Cu]Cu-NODAGA-αSTn-IgG4 TM were intravenously injected into a lateral tail vein of the mice. Imaging was performed using dynamic scans acquired over 2 h with a small animal PET scanner (microPET P4, Siemens) at time point 0 h, and over 1 h at the time points 7, 20, 47 and 66 h after TM injection. Standard uptake values (SUV) defined as tissue concentration (MBq/mL)/injected dose (MBq)/body weight (g) in (g/ml) were used to express activity concentrations. Images were visualized and quantified using ROVER software (ABX).

### Statistical analysis

Statistical significance was determined using GraphPad Prism software 7.0 (GraphPad Software Inc.). Statistical tests were specified in the respective figure legends. *P* values below 0.05 were considered significant as follows: *p < 0.1; **p < 0.01; ***p < 0.001 and ****p < 0.0001. All error bars are represented either as standard error of the mean (SEM) or standard deviation (SD).

## Results

### Construction of a novel IgG4-based TM targeting the STn antigen

As previously described, UniCAR T-cells have been successfully redirected using TMs with different formats, such as nanobody- and scFv-based TMs, to target several tumor antigens [8–11]. In this work the possibility of producing and using a TM with increased size to redirect UniCAR T-cells was explored. Hence, a novel IgG4-based TM format targeting STn was constructed (Fig. 1). The structure of this construct is similar to the scFv-based TM with the additional insertion of the hinge and Fc (C_H_2-C_H_3) regions derived from human IgG4 antibodies. These regions were introduced between the binding domain (scFv) derived from the αSTn mAb L2A5 [28] at the N-terminus, and the E5B9 epitope used for UniCAR T-cell recognition (Fig. 1b). A LP sequence was added N-terminally to promote secretion of the TM into the cell culture supernatant and a His-tag was fused at the C-terminus to allow TM detection. The entire sequence encodes one polypeptide chain and given that the cysteine residues present in the hinge region will form disulfide bridges, a secreted homodimer composed of two identical polypeptide chains is produced. This molecule resembles the format of an IgG4 antibody and has a molecular weight (MW) of around 111 kDa, which is considerably increased compared to a scFv-based TM (35 kDa). Additionally, and based on the peptide tag E7B6 incorporated in the extracellular part of the UniCAR, cell surface expression on T-cells is routinely verified prior to performing the assays, as exemplified in Fig. 1c. Noteworthy, UniCAR expression directly correlates with the expression of co-translated EGFP marker protein.

### Expression, purification and characterization of the αSTn-IgG4 TM

**Fig. 2:**
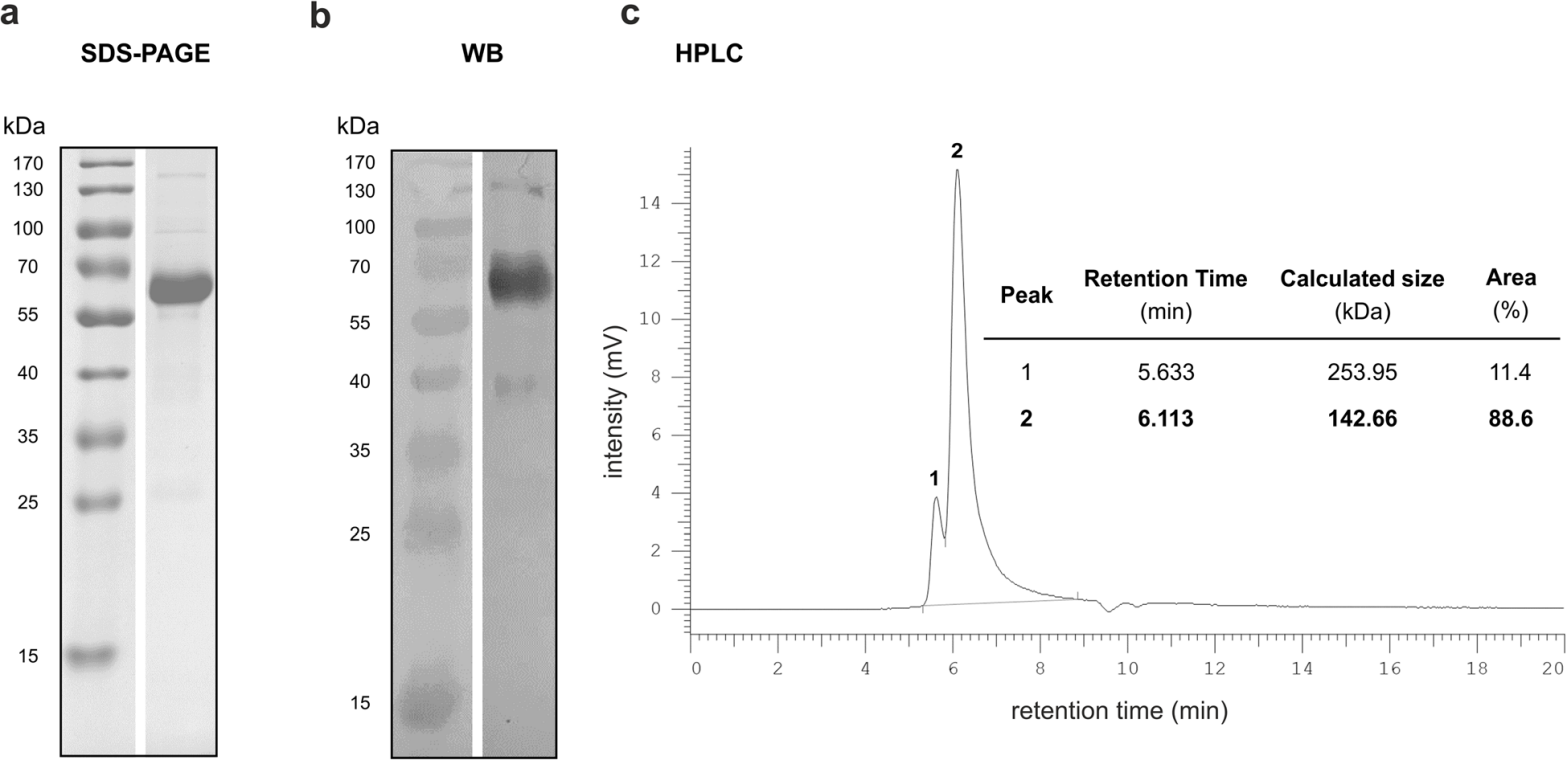
Analysis of purified αSTn-IgG4 TM by SDS-PAGE, Western Blot and size exclusion HPLC. The αSTn-IgG4 TM was purified from the supernatant of TM-producing 3T3 cells using protein A columns. The dialyzed TM was resolved by SDS-PAGE and stained with **(a)** Coomassie Brilliant Blue G250 or **(b)** analyzed by WB using αHis mAb for detection of the C-terminus His-tag. **c** Chromatogram obtained by size exclusion HPLC of 10 μg of purified TM

The open reading frame of the αSTn-IgG4 TM was cloned into the p6NST50 vector which was used for transduction of murine 3T3 cells. The resulting cell line served for production of the TM. Purification from cell culture supernatants was performed using protein A affinity chromatography. The purified αSTn-IgG4 TM was analyzed by SDS-PAGE, immunoblotting and size exclusion HPLC to confirm the correct molecular weight and purity. Given that the purified STn-IgG4 TM forms a homodimer, a MW of 111 kDa is calculated for this molecule. However, due to the denaturing conditions of the SDS-PAGE, the disulfide bridges within the hinge region are reduced and αSTn-IgG4 monomers are expected to be observed with a theoretical MW of 55 kDa. As shown in both the SDS-PAGE and WB analyses, a major band with a MW of around 60 kDa corresponding to the αSTn-IgG4 monomers is observed (Fig. 2a and b). Moreover, a faint band with a MW of around 130 kDa was obtained, most likely representing the homodimeric conformation of the αSTn-IgG4 TM. Size exclusion HPLC was used to further confirm the purity and size of the TM under native conditions. As expected, a major peak with 89% of the total area was observed with a MW of 143 kDa, corresponding to the homodimer αSTn-IgG4 TM (Fig. 2c). Additionally, a minor peak (11% of the total area) was obtained at a MW of 254 kDa, which suggests the presence of αSTn-IgG4 oligomers or possible contaminants (Fig. 2c). Collectively, these results demonstrate the successful production and purification of the homodimeric αSTn-IgG4 TM with high purity for further in vitro and in vivo functional characterization.

### Binding and affinity assessment of the αSTn-IgG4 TM

**Fig. 3:**
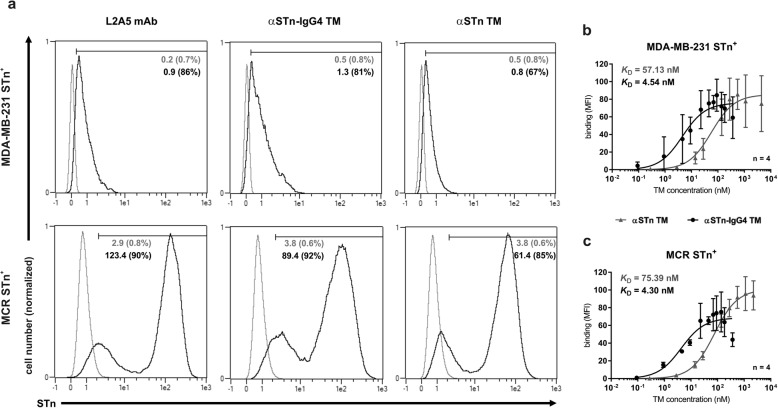
Binding of αSTn and αSTn-IgG4 TMs to STn-expressing cancer cells. **a** Both MDA-MB-231 and MCR cancer cells expressing STn were stained with 1 μg of TMs, mAb 5B9 and PE-labeled anti-mouse-IgG mAb. Staining with αSTn mAbs L2A5 and αSTn TM served as positive controls. L2A5 mAb was detected using an Alexa Fluor 488 anti-mouse IgM mAb. Stained cells (black lines) and respective controls (grey lines) are displayed as histograms. MFI and percentage of positively stained cells (in parenthesis) are shown. Results for one representative binding assay are depicted. **b** and **c** Binding affinity curves of αSTn (grey) or αSTn-IgG4 (black) TMs to MDA-MB-231 STn^+^**(b)** and MCR STn^+^**(c)** cells were obtained using increasing concentrations of TM. Binding was detected as described above. Data are presented as MFI average values ± SD from four independent experiments

The first feature assessed was the capacity of the novel αSTn-IgG4 TM to specifically bind to STn-expressing cancer cells. This was done by flow cytometry using the breast cancer cell line MDA-MB-231 STn^+^ and the bladder carcinoma cell line MCR STn^+^. The parental antibody L2A5 mAb and the scFv-based TM (αSTn TM) were used as positive controls. As represented in Fig. 3a, αSTn-IgG4 TM has a comparable binding pattern to both positive controls. Additionally, no binding was observed when performing an equivalent staining on the corresponding cancer cell lines lacking the expression of STn (WT cell lines) (Fig. S1). Binding affinity curves of both αSTn TMs were obtained for STn-expressing cell lines by TM titration (Fig. 3b and c). Affinity binding values (*K*_D_ values) of around 4 nM were obtained for the αSTn-IgG4 TM, in contrast to 57 and 75 nM determined for the αSTn TM. This observation further corroborates the principle that more binding valences increase the binding strength of antibody derivatives. As the αSTn-IgG4 TM has two αSTn binding arms in comparison to the monovalent αSTn TM, it binds more effectively to STn-expressing tumor cells. The acquired data additionally show that after binding of the TM to the target antigen, the UniCAR tag (E5B9 epitope) is still accessible for recognition by the αE5B9 UniCAR domain, which is an important requirement for successful interaction with UniCAR T-cells. Overall, these results demonstrate that the αSTn-IgG4 TM is able to specifically bind its target antigen on the surface of different cancer cell lines with an increased binding affinity in comparison to the previously designed αSTn TM.

### Engagement via αSTn-IgG4 TM leads to specific lysis of STn^+^ tumor cells by UniCAR T-cells

**Fig. 4:**
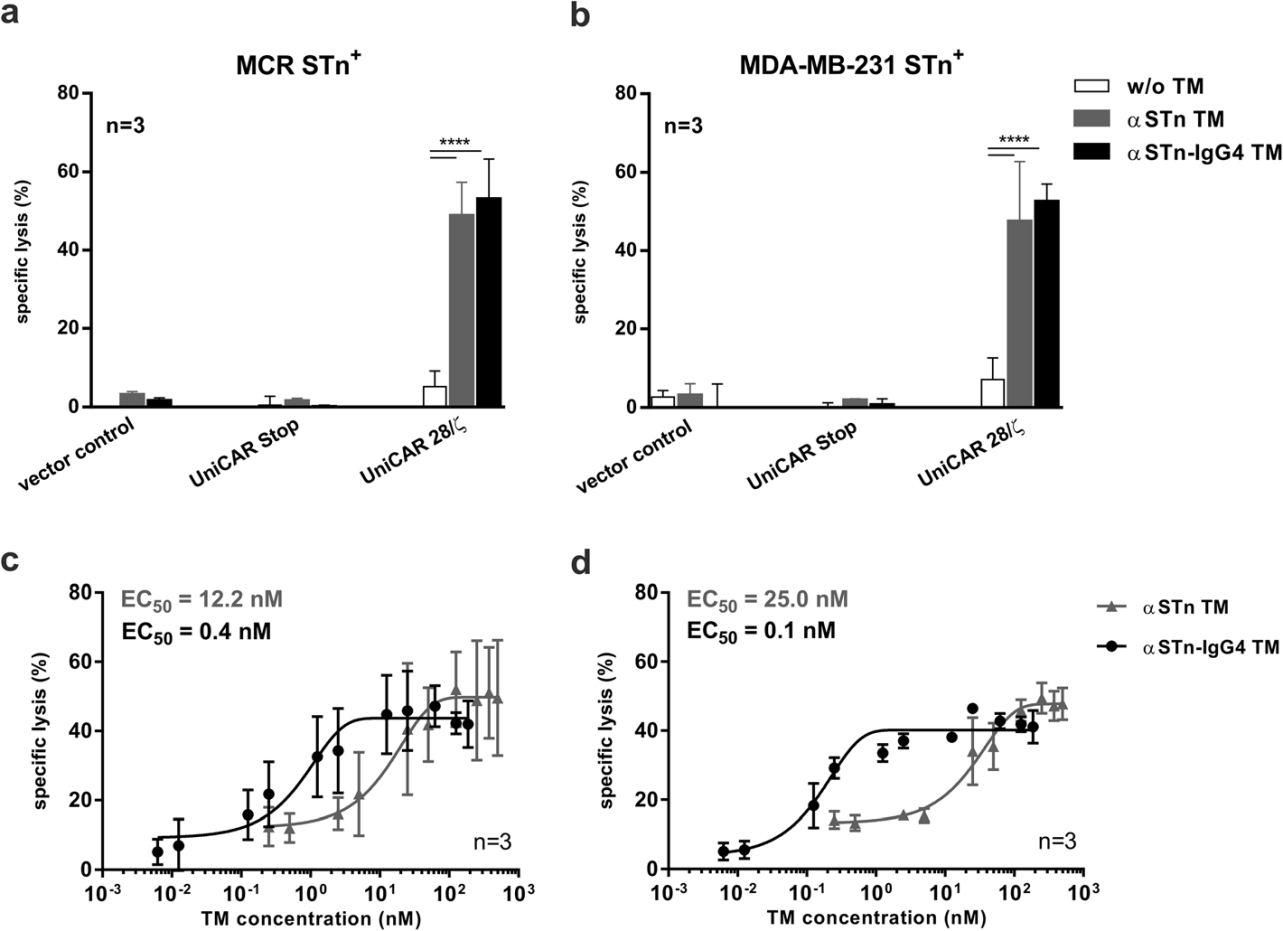
Cytotoxic potential of retargeted UniCAR T-cells via αSTn-IgG4 TM. Killing of MDA-MB-231 STn^+^**(b)** and MCR STn^+^**(a)**, was determined using standard chromium release assay. Chromium-labeled STn-expressing cell lines were incubated with control T-cells (vector control and UniCAR Stop) or UniCAR 28/ζ T-cells for 24 h at an E:T ratio of 5:1, in the presence or absence of 10 pmol αSTn TM and αSTn-IgG4 TM. **c** and **d** Following a similar experimental setup using increasing TM concentrations, half maximal effective concentration (EC_50_) was determined from the resulting dose-response curves using MDA-MB-231 STn^+^**(c)** and MCR STn^+^**(d)** cells. Data for three individual donors were summarized as mean specific lysis ± SD. Statistical significance was obtained using 2-way ANOVA with Bonferroni multiple-comparison test (****p < 0.0001, with respect to w/o TM experimental setting)

After confirming the specific binding of the αSTn-IgG4 TM to STn-expressing cancer cells, functionality regarding in vitro killing efficiency was assessed using chromium release assays. For that, human T-cells from healthy donors were transduced with lentiviral vectors encoding either the UniCAR sequence comprising an intracellular signaling domain (UniCAR 28/ζ), the UniCAR sequence lacking any signaling domains (UniCAR Stop) or the EGFP marker protein alone (vector control). UniCAR Stop and vector control T-cells served as negative controls. Chromium release was measured 24 h after co-culture of STn-expressing cancer cells with transduced T-cells at an E:T ratio of 5:1 in the presence or absence of αSTn-IgG4 TM or αSTn TM. As shown in Fig. 4a and b, both TMs induce efficient lysis of STn-expressing tumor cells by UniCAR 28/ζ T-cells. Contrarily, UniCAR T-cells were not able to eradicate tumor cells in the absence of TM. As expected, UniCAR Stop and vector control T-cells were completely inert in the presence or absence of TM. Using a similar assay set up, the αSTn-IgG4 TM was titrated in order to calculate the EC_50_. As represented in Fig. 4c and d, EC_50_ values of 0.4 nM and 0.1 nM were determined for MDA-MB-231 STn^+^ and MCR STn^+^ cells using the αSTn-IgG4 TM, respectively. In comparison to the αSTn TM (12.2 nM and 25.0 nM), a lower concentration of αSTn-IgG4 TM was sufficient to achieve a similar maximal tumor cell lysis. Most likely, the increased avidity results in higher killing efficiency of the αSTn-IgG4 TM. Taken together, these data show that UniCAR T-cells can be redirected using an IgG4-based TM to specifically target STn-expressing cancer cells with increased efficiency.

### UniCAR T-cells redirected by the αSTn-IgG4 TM promote the release of proinflammatory cytokines

**Fig. 5:**
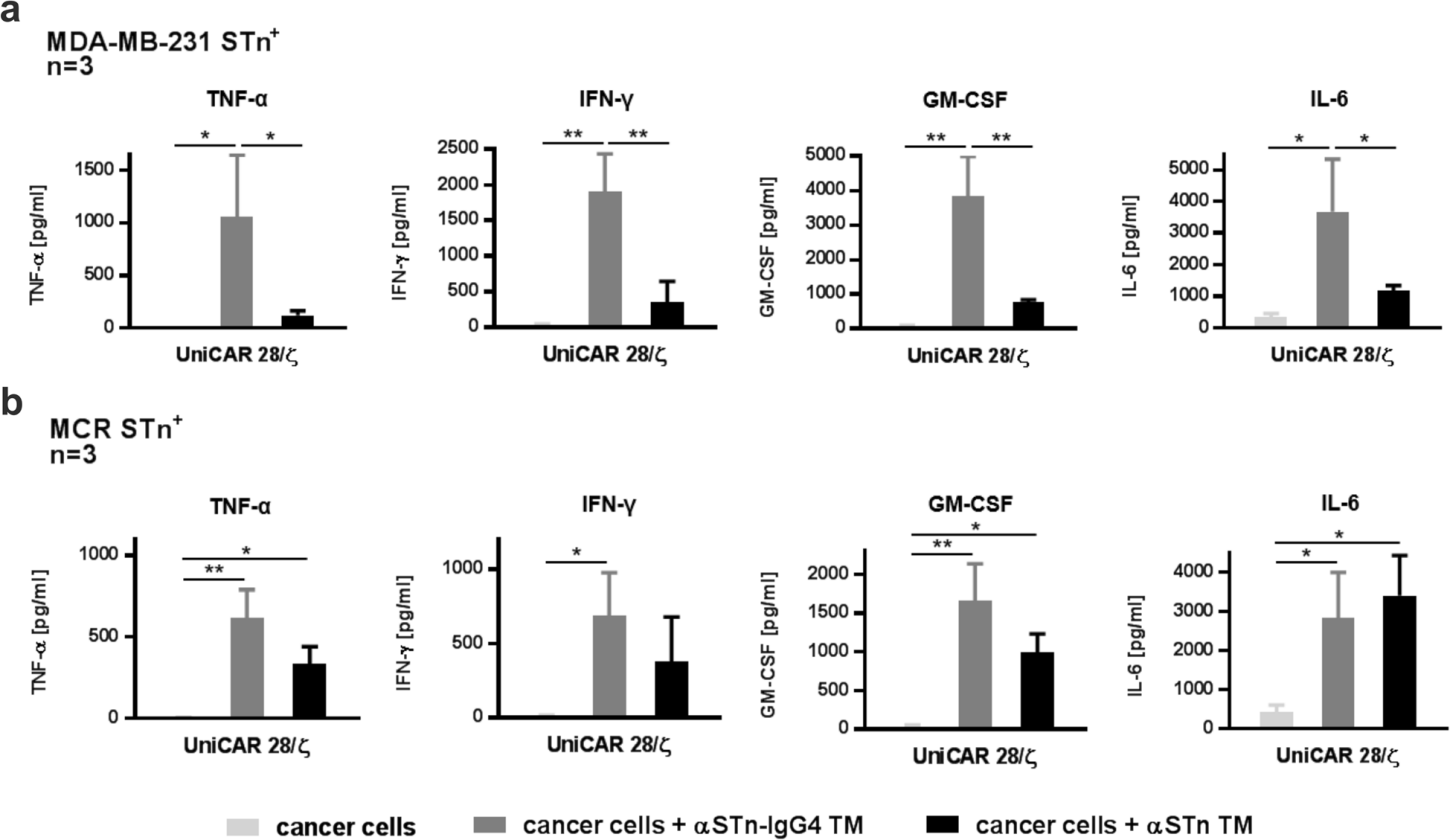
Proinflammatory cytokine secretion profile of UniCAR T-cells redirected via αSTn and αSTn-IgG4 TMs. T-cells transduced with UniCAR 28/ζ constructs were co-cultured with MDA-MB-231 STn^+^**(a)** or MCR STn^+^**(b)** in the presence or absence of αSTn or αSTn-IgG4 TMs at an E:T ratio of 5:1. After incubation for 24 h, cell culture supernatants were harvested and analyzed using the MACSPlex Cytokine 12 kit. Average cytokine concentrations and SD for three individual donors are shown. Statistical significance was determined using 1-way ANOVA with Bonferroni multiple-comparison test (*p < 0.05; **p < 0.01)

Having substantiated the TM-mediated killing capacity of UniCAR T-cells upon cross-linkage to STn-expressing tumor cells using the αSTn-IgG4 TM, the cytokine release pattern of UniCAR T-cells was assessed. For that, UniCAR T-cells were incubated in the presence or absence of MDA-MB-231 STn^+^ (Fig. 5a) or MCR STn^+^ (Fig. 5b) cells, with or without the αSTn or αSTn-IgG4 TMs. After 24 h of incubation, the concentration of cytokines present in cell-free culture supernatants was analyzed using a multiplex assay (MACSPlex Cytokine 12 Kit). In this assay, detection and quantification of the cytokines TNF-α, IFN-γ, IFN-α, GM-CSF, IL-2, IL-4, IL-5, IL-6, IL-9, IL-10, IL-12 and IL-17A are performed simultaneously. Significant cytokine concentrations were detected for TNF-α, IFN-γ, GM-CSF and IL-6 (Fig. 5). Except for IL-6, all of these cytokines were specifically detected only in co-cultures of UniCAR T-cells with STn^+^ cancer cells in the presence of αSTn or αSTn-IgG4 TMs, with a significantly higher amount of secreted cytokines for the αSTn-IgG4 TM in comparison to the αSTn TM. Nevertheless, IL-6 was detected in all conditions that include cancer cells, suggesting that IL-6 is intrinsically secreted by the STn-expressing cancer cell lines used. This is in accordance with similar results previously observed for the αSTn TM using the same cell lines [12]. Importantly, none of the tested cytokines were present in supernatants recovered from negative control UniCAR T-cells (UniCAR Stop and vector control) or from UniCAR T-cells in the absence of TM and cancer cells (Fig. S2). Overall, the release of proinflammatory cytokines was specifically observed for the combination of UniCAR T-cells in the presence of target cancer cells and αSTn-IgG4 TM.

### Efficient and TM-dependent inhibition of tumor growth in experimental mice by UniCAR T-cells redirected with the αSTn-IgG4 TM

**Fig. 6:**
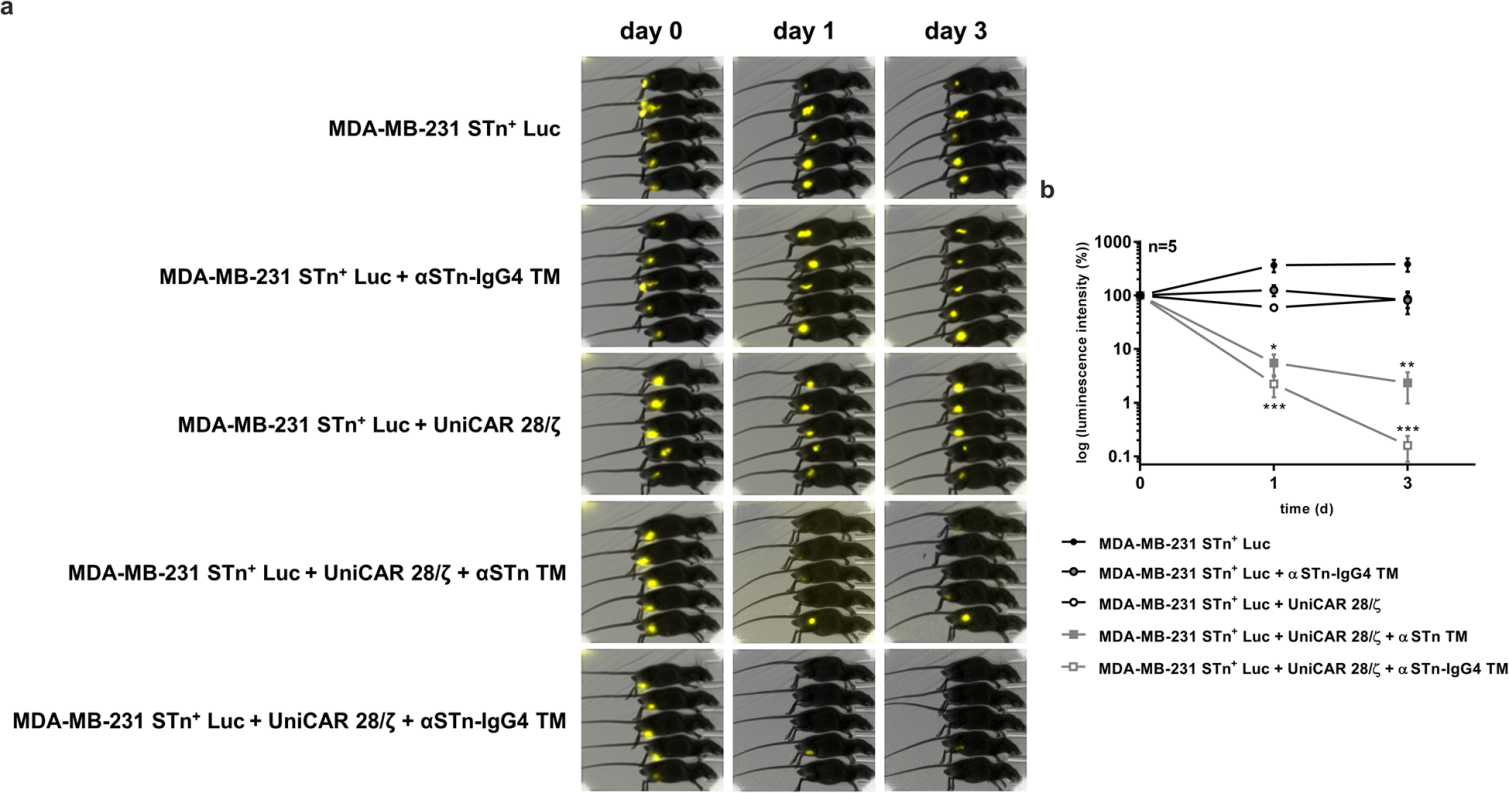
In vivo killing capacity of redirected UniCAR T-cells in NOD-SCID mice using αSTn-IgG4 and αSTn TMs. Five groups composed of five NOD-SCID mice were established, from which mice injected with MDA-MB-231 STn^+^ Luc tumor cells alone; tumor cells mixed with αSTn-IgG4 TM or tumor cells mixed with UniCAR 28/ζ T-cells were used as negative controls. Mice co-injected with tumor cells, UniCAR 28/ζ T-cells and αSTn TM or αSTn-IgG4 TM were used as treatment groups. All injections were performed subcutaneously into the right hind flank. **a** Bioluminescence imaging of anesthetized mice was performed at day 0, 1 and 3. **b** Based on the luminescence imaging results obtained, a quantitative analysis was performed. The values were normalized to the initial measurement performed after injection on day 0 and represented as mean ± SEM. Statistical significance was determined using two-way ANOVA with Bonferroni multiple-comparison test with respect to the control group injected with MDA-MB-231 STn^+^ Luc and UniCAR 28/ζ T-cells (*p < 0.1; **p < 0.01; ***p < 0.001)

Once killing efficiency was validated in vitro, the in vivo anti-tumor activity of UniCAR T-cells retargeted via the αSTn-IgG4 TM was analyzed using a xenograft tumor mouse model. For that, MDA-MB-231 STn^+^ cells transduced with luciferase (MDA-MB-231 STn^+^ Luc cells) were required to allow tumor imaging and follow-up during the experiment. Consequently, five groups of NOD-SCID mice were created. Three groups were injected subcutaneously with MDA-MB-231 STn^+^ Luc cells alone, MDA-MB-231 STn^+^ Luc cells with αSTn-IgG4 TM or MDA-MB-231 STn^+^ Luc cells with UniCAR T-cells, serving as controls. For the two treatment groups, MDA-MB-231 STn^+^ Luc cells with UniCAR T-cells and αSTn TM or αSTn-IgG4 TM were injected. Tumor growth was monitored over 3 days by bioluminescence imaging. Qualitative imaging and corresponding quantitative results are shown in Fig. 6a and b, respectively. Corroborating the in vitro data, injection of STn^+^ target cells with UniCAR 28/ζ T-cells and αSTn or αSTn-IgG4 TMs significantly inhibited tumor growth already 1 day after injection. Absence of tumors was further verified up to 3 days after injection. Conversely and as expected for the control groups, injection of UniCAR T-cells or αSTn-IgG4 TM alone did not hinder tumor growth. The quantitative analysis of the imaging results reveals an increased killing potential of αSTn-IgG4-redirected UniCAR T-cells compared to the αSTn TM, represented by a more pronounced decrease in tumor bioluminescence intensity obtained upon injection of the αSTn-IgG4 TM (Fig. 6b). These results demonstrate the effective and TM-dependent eradication of STn-expressing tumors by redirected UniCAR T-cells in vivo, with significant inhibition of tumor growth using the αSTn-IgG4 TM.

### Kinetics and PET imaging of the αSTn-IgG4 TM in mice bearing STn^+^ tumors

**Fig. 7:**
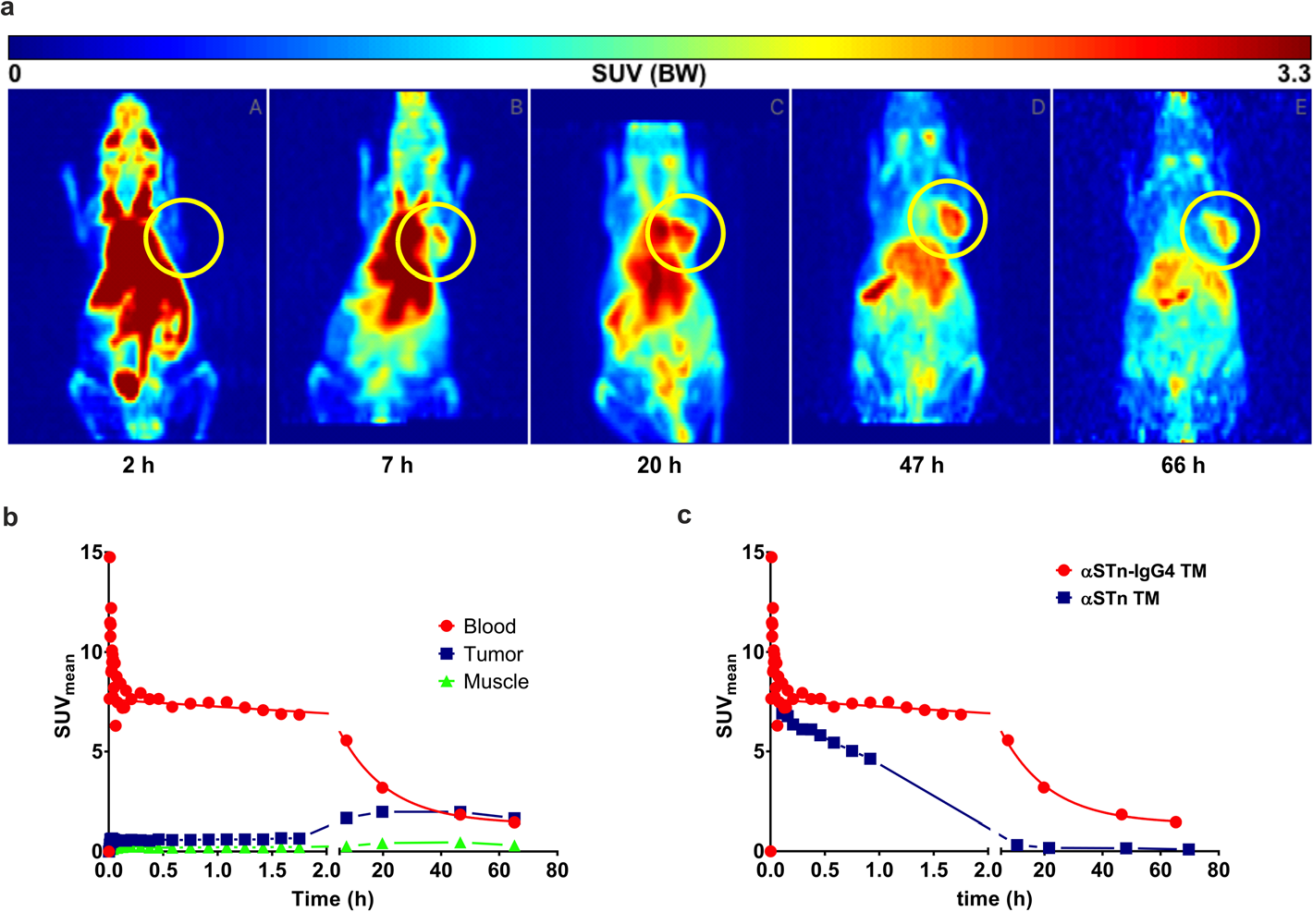
PET analysis of STn^+^ tumor-bearing mouse injected with [^64^Cu]Cu-NODAGA-αSTn-IgG4 TM. The TMs were conjugated with NODAGA, labeled with copper-64 (^64^Cu) and injected as a single bolus intravenously into NOD-SCID mice bearing established MDA-MB-231 STn^+^ Luc tumors. **a** Representative coronal view of the maximum intensity projection (MIP) of the [^64^Cu]Cu-NODAGA-αSTn-IgG4 TM distribution after 2, 7, 20, 47 and 66 h. Tumor is highlighted by yellow circles. **b** Time activity curves (TAC) of the dynamic PET analysis of regions of interest (ROI) obtained using the radiolabeled αSTn-IgG4 TM for blood, tumor and muscle. **c** Comparative view of blood TAC for the αSTn-IgG4 (red curve) and αSTn TMs (blue curve) over 66 h. SUV, standard uptake values

To evaluate the pharmacokinetics of the radiolabeled αSTn-IgG4 TM for in vivo imaging of STn-overexpressing tumor sites, the αSTn-IgG4 TM was conjugated with the chelator NODAGA and further labeled with the PET isotope copper resulting in [^64^Cu]Cu-NODAGA-αSTn-IgG4 TM. A labeling yield and radiochemical purity (decay-corrected) of 94 ± 5% was determined by radio-ITLC. Furthermore, a molar activity of 11 ± 8 GBq/μmol was obtained. For comparison, the αSTn TM was conjugated with NODAGA and labeled with copper following a similar protocol. To perform small animal PET, MDA-MB-231 STn^+^ Luc tumors were established on the right shoulder of NOD-SCID mice 2 weeks prior to injection of the radiolabeled TMs. Figure 7a summarizes accumulation at the tumor site and clearance of the [^64^Cu]Cu-NODAGA-αSTn-IgG4 TM over 3 days. As shown, a maximum accumulation at the tumor site was observed around 46 h after injection with a slow blood clearance between 50 to 66 h (Fig. 7a and b). These data indicate that the αSTn-IgG4 TM has a delayed maximum accumulation at the tumor compared to the αSTn scFv-based TM (around 2 h). Additionally, a longer lasting accumulation is obtained in contrast to the scFv-based TM. Furthermore, time activity curves of the dynamic PET analysis of blood were used to determine serum half-life (Fig. 7c). Based on the values of the area-under-curve obtained for the [^64^Cu]Cu-NODAGA-αSTn TM and [^64^Cu]Cu-NODAGA-αSTn-IgG4 TM (40.3 and 200.2, respectively), blood half-life values of 1.2 h for the αSTn TM and 12 h for the αSTn-IgG4 TM were calculated. In summary, the obtained results demonstrate a specific and long-lasting accumulation of the αSTn-IgG4 TM at the tumor site in accordance with its extended half-life.

## Discussion

Recently, switchable adapter CAR T-cell platforms have been established to circumvent the main hurdles of conventional CAR T-cell therapies [5]. Thereby, activity of CAR T-cells is controlled based on the specificity, size, molecular design and pharmacokinetics of the selected CAR adaptor. Our group established the modular UniCAR platform, in which various TMs with short half-life targeting different tumor-associated antigens have been demonstrated to redirect UniCAR T-cells to specifically eradicate tumor cells [8–12]. As a key prerequisite of the UniCAR system, TMs should be rapidly eliminated from the peripheral blood to provide a quick steering and self-limiting safety switch in case severe side effects arise. Consequently, activation and potential adverse effects of UniCAR T-cells can be easily tuned by dosing TM infusions. To overcome the continuous infusion required for such small-sized TMs at later stages of therapy, we developed an extended half-life TM targeting the STn antigen based on the backbone of human IgG4 antibodies. For that, the scFv derived from the mAb L2A5 and the E5B9 epitope were fused to the human IgG4 hinge and Fc region. The backbone of the IgG4 molecule was chosen due to the limited binding to Fc receptors as well as the weak or absent activation of both complement and antibody-dependent cellular cytotoxicity [29]. As a result, IgG4 antibodies have been widely used to design therapeutic antibodies when a weak or absent immune cell activation is desired, yet maintaining an increased plasmatic half-life comparable to the other IgG subclasses [30].

We successfully produced a homodimeric αSTn-IgG4 TM with considerably high purity that specifically binds to STn-expressing cancer cell lines and promotes efficient tumor cell lysis by redirected UniCAR T-cells both in vitro and in experimental mice. Both scFv-based and IgG4-based TMs bind to STn^+^ cancer cells with high affinity. Bearing in mind that the underlying anti-STn mAb L2A5 is a dekavalent IgM mAb [28], the high calculated affinities of these TMs were surprising. Comparing both TMs, a 12 to 17-fold increase in binding affinity was obtained for the αSTn-IgG4 TM (4 nM compared to 57 nM and 75 nM). This result can be mainly explained by the fact that the αSTn-IgG4 TM has two binding sites in contrast to the monovalent αSTn TM. Furthermore, lower amounts of IgG4-based TM were required to obtain similar specific cell lysis compared to the scFv-based TM. This may again be explained due to the geometry and valency of the αSTn-IgG4 TM. Emphasis should also be placed to the importance of the distance and molecular geometry of the immunological synapse to ensure adequate activation of effector cell functions. Previous studies using conventional CARs have reported attenuated activation signaling due to the formation of longer immunological synapses and variations in the antigen location and density on target cells [31, 32]. Interestingly, recent reports highlight the effective formation of the immunological synapse and consequent T cell activation using different modular CAR formats, demonstrating a surprising plasticity and variability of such approaches [3, 33, 34]. Here, we demonstrate that the immunological synapse formed by the large αSTn-IgG4 TMs does not affect the activation nor cytotoxic potential of UniCAR T-cells targeting the STn antigen. This validates the versatility of the UniCAR system, which can be easily combined with TM formats of different sizes and designs without hampering UniCAR T-cell activation and killing efficiency. Accordingly, upon TM-mediated cancer cell lysis, UniCAR T-cells secreted the cytokines TNF-α, IFN-γ, GM-CSF and IL-2. Worth mentioning, IL-6, which is mainly held responsible for cytokine release syndrome, was detected in supernatants of samples containing target cells regardless of the presence or absence of the αSTn-IgG4 TM. This suggests that IL-6 is released by MDA-MB-231 and MCR cancer cells and not by activated UniCAR T-cells, which is in line with our previous publication and other studies [12, 35, 36]. As stated before, proinflammatory cytokines are specifically released following TM-dependent cross-linkage of tumor and UniCAR T-cells. TNF-α, IFN-γ and GM-CSF are known to be involved in mechanisms such as induction of tumor cell apoptosis as well as proliferation and activation of T-cells. The aforementioned cytokines can further stimulate other immune cells to overcome the immunosuppressive microenvironment often seen in solid cancers, including STn^+^ tumors [37]. Moreover, STn has been recently reported to be expressed on the surface of infiltrating myeloid derived suppressor cells (MDSCs) [37]. Hence, in a clinical setting eradication of STn-expressing tumors via redirected UniCAR T-cells may be augmented by the additional depletion of immune-suppressive MDSCs, which subsequently endorses immune re-engagement.

PET imaging results show a specific enrichment of the αSTn-IgG4 TM at the tumor site with maximum accumulation at around 46 h after injection and a slow clearance between 50 to 66 h. Furthermore, dynamic PET analysis emphasizes the extended blood half-life of 12 h for the αSTn-IgG4 TM compared to approximately 1 h obtained for the αSTn TM. These properties of the αSTn-IgG4 TM are mainly related to its increased molecular weight, which leads to a delayed but prolonged accumulation at the tumor, additionally preventing its rapid clearance via the kidneys. FcRn-mediated recycling may also play a role and affect the lifespan of the αSTn-IgG4 TM, increasing the retention time of this molecule in the plasma [38]. In light of future clinical applications, combination of small-sized and extended half-life TMs with UniCAR T-cells might allow a more convenient treatment regimen for cancer patients. During onset of therapy when tumor burden is high, an scFv-based TM should be continuously applied to enable a rapid off-switch of UniCAR T-cells by ceasing TM infusion in case of unwanted toxicities. In later stages of therapy, when tumor burden is lower and thus side effects are less likely to occur, the use of a TM with extended half-life may be important to reduce the number of infusions as well as to enhance eradication of residual tumor cells. Such an approach may be particularly relevant when targeting STn-expressing tumors, as STn expression is frequently associated with tumor invasiveness, metastasis and immune escape, especially in breast and bladder cancer [17, 39–41]. Even though the blood clearance of the IgG4-based TM is considerably delayed, the safety profile is not compromised since the TM would be eliminated at some point and therefore, a controlled inactivation of UniCAR T-cells would be achieved. In this way, the risk to develop long-term side effects caused by continuously activated conventional CAR T-cells would still be minimized.

The pharmacokinetic features and stable accumulation of radiolabeled αSTn-IgG4 TMs at the tumor site combined with the possibility to simultaneously redirect UniCAR T-cells to eradicate tumor cells further highlights its potential application for theranostic purposes. Thus, therapy and tumor imaging could be combined in a single radiolabeled TM aiming at a patient-centered care. Depending on the selected radionuclide, the modified TM might be routinely used for PET/CT imaging or endoradiotherapy. In that way, the radiolabeled αSTn-IgG4 TM could be used as a tracer for diagnosis and monitoring of tumor diseases while enabling simultaneous immunotherapy with the UniCAR system. Yet, additional work and validation are required in this regard.

## Conclusions

Taken together, a novel IgG4-based TM with extended half-life targeting the STn antigen has been developed and proven to efficiently redirect UniCAR T-cells to STn-expressing cancer cells in a highly target-specific manner both in vitro and in vivo. Thereby, the UniCAR T-cell technology can be used in a customized way, in which TMs with different pharmacokinetic features, and if required with different specificities, can be administered according to the stage and demands observed during cancer therapy. Contrarily to the current conventional CAR T-cell therapies, this type of tailored approach would provide a more convenient and controllable way to treat patients according to the therapy stage and response to treatment. Furthermore, radiolabeled TMs may be attractive candidates for diagnostics and therapy monitoring. Their high potential for theranostic applications fosters the transition from conventional to personalized medicine.

## Supplementary information

**Additional file 1: Figure S1.** Binding of αSTn and αSTn-IgG4 TMs to MDA-MB-231 and MCR wild-type (WT) cancer cells. Both MDA-MB-231 and MCR WT cancer cells were stained with 1 μg of TMs, mAb 5B9 and PE-labeled anti-mouse-IgG mAb. The αSTn L2A5 mAb was detected using an Alexa Fluor 488 anti-mouse IgM mAb. Stained cells (black lines) and respective isotype controls (grey lines) are displayed as histograms. MFI values are shown. Results for one representative binding assay are shown. **Figure S2.** Cross-linkage of UniCAR T-cells with STn^+^ tumor cells via αSTn-IgG4 TM results in release of cytokines. In a 24 h-cytokine-release assay, **(a)** MDA-MB-231 STn^+^ or **(b)** MCR STn^+^ cells were incubated with vector control, UniCAR Stop or UniCAR 28/ζ T-cells in the presence or absence of αSTn-IgG4 TM (E:T ratio of 5:1). Cytokine concentrations in cell-free co-culture supernatants were detected using the MACSPlex Cytokine 12 kit. Average cytokine concentrations and SD for three individual donors are shown. Statistical significance was determined using 2-way ANOVA with Bonferroni multiple-comparison test (**p < 0.01; ***p < 0.001 and ****p < 0.0001).

## Data Availability

Data confirming the results of this study are presented in the manuscript and are available from the corresponding author upon reasonable request.

## Abbreviations

CAR: Chimeric antigen receptor
C_H_2: Constant domain 2 of the heavy chain of a monoclonal antibody
C_H_3: Constant domain 3 of the heavy chain of a monoclonal antibody
DMEM: Dulbecco’s modified Eagle’s medium
EC_50_: Half maximal effective concentration
EDTA: Ethylenediamine tetraacetic acid
EGFP: Enhanced green fluorescent protein
ELISA: Enzyme-linked immunosorbent assay
E:T: Effector to target cells
FBS: Fetal bovine serum
IgG4: Immunoglobulin 4
*K*_D_: Equilibrium dissociation constant
Luc: Firefly luciferase
mAb: monoclonal antibody
MDSCs: Myeloid derived suppressor cells
MFI: Median fluorescence intensities
MW: Molecular weight
NOD-SCID: Non-obese diabetic-severe combined immune-deficient
PBMCs: Peripheral blood mononuclear cells
PBS: Phosphate buffered saline
PET: Positron emission tomography
CT: Computed tomography
radio-ITLC: radio thin-layer chromatography
scFv: single-chain variable fragment
SD: Standard deviation
SDS-PAGE: Sodium dodecyl sulfate polyacrylamide gel electrophoresis
SE-HPLC: Size exclusion high-performance liquid chromatography
STn: Sialyl-Tn
SUV: Standard uptake values
TM: Target module
UniCAR: Universal CAR
V_H_: Variable domain of the heavy chain of a monoclonal antibody
V_L_: Variable domain of the light chain of a monoclonal antibody
WB: Western blot
