# Supplementary material for: Extended half-life target module for sustainable UniCAR T-cell treatment of STn-expressing cancers
Source: J Exp Clin Cancer Res. 2020 May 5;39:77. doi: 10.1186/s13046-020-01572-4 (PMC7201957; doi:10.1186/s13046-020-01572-4)
Supplement: Supplementary file 1 — Additional file 1: Figure S1. Binding of αSTn and αSTn-IgG4 TMs to MDA-MB-231 and MCR wild-type (WT) cancer cells. Both MDA-MB-231 and MCR WT cancer cells were stained with 1 μg of TMs, mAb 5B9 and PE-labeled anti-mouse-IgG mAb. The αSTn L2A5 mAb was detected using an Alexa Fluor 488 anti-mouse IgM mAb. Stained cells (black lines) and respective isotype controls (grey lines) are displayed as histograms. MFI values are shown. Results for one representative binding assay are shown. Figure S2. Cross-linkage of UniCAR T-cells with STn+ tumor cells via αSTn-IgG4 TM results in release of cytokines. In a 24 h-cytokine-release assay, (a) MDA-MB-231 STn+ or (b) MCR STn+ cells were incubated with vector control, UniCAR Stop or UniCAR 28/ζ T-cells in the presence or absence of αSTn-IgG4 TM (E:T ratio of 5:1). Cytokine concentrations in cell-free co-culture supernatants were detected using the MACSPlex Cytokine 12 kit. Average cytokine concentrations and SD for three individual donors are shown. Statistical significance was determined using 2-way ANOVA with Bonferroni multiple-comparison test (**p < 0.01; ***p < 0.001 and ****p < 0.0001). [file 13046_2020_1572_MOESM1_ESM.docx]

**Additional File 1**


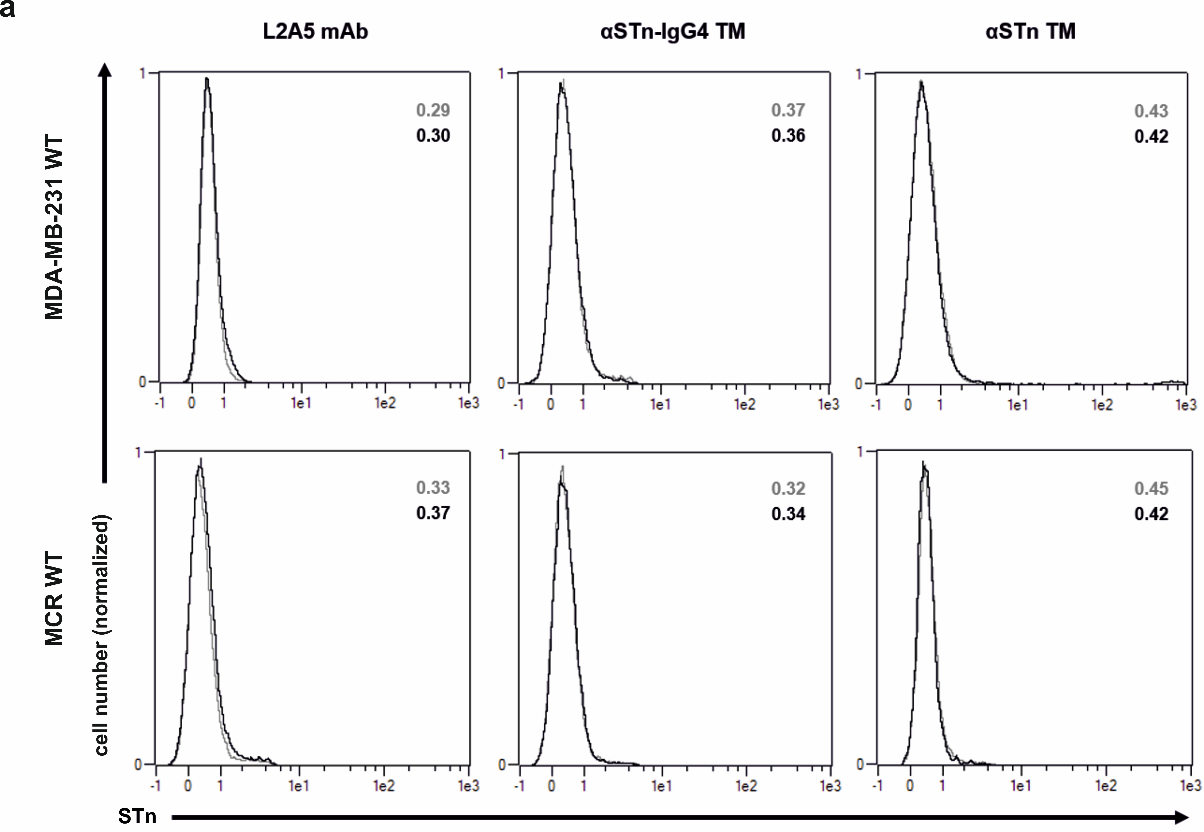


**Fig. S1 - Binding of αSTn and αSTn-IgG4 TMs to MDA-MB-231 and MCR wild-type (WT) cancer cells.** Both MDA-MB-231 and MCR WT cancer cells were stained with 1 µg of TMs, mAb 5B9 and PE-labeled anti-mouse-IgG mAb. The αSTn L2A5 mAb was detected using an Alexa Fluor 488 anti-mouse IgM mAb. Stained cells (black lines) and respective isotype controls (grey lines) are displayed as histograms. MFI values are shown. Results for one representative binding assay are shown.


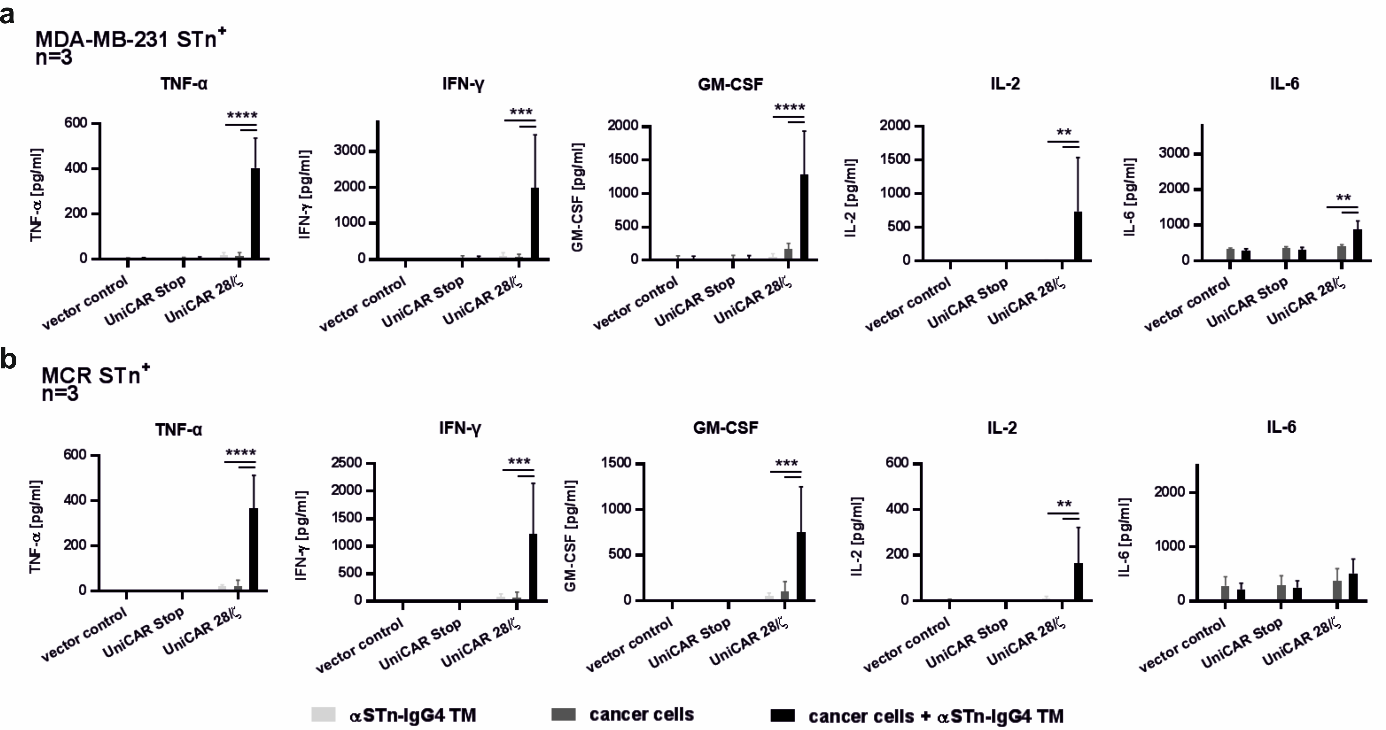


**Fig. S2 - Cross-linkage of UniCAR T-cells with STn^+^ tumor cells via αSTn-IgG4 TM results in release of cytokines.** In a 24 h-cytokine-release assay, **(a)** MDA-MB-231 STn^+^ or **(b)** MCR STn^+^ cells were incubated with vector control, UniCAR Stop or UniCAR 28/ζ T-cells in the presence or absence of αSTn-IgG4 TM (E:T ratio of 5:1). Cytokine concentrations in cell-free co-culture supernatants were detected using the MACSPlex Cytokine 12 kit. Average cytokine concentrations and SD for three individual donors are shown. Statistical significance was determined using 2-way ANOVA with Bonferroni multiple-comparison test (**p<0.01; ***p<0.001 and ****p<0.0001).
